# Supplementation with active vitamin D3 ameliorates experimental autoimmune thyroiditis in mice by modulating the differentiation and functionality of intrathyroidal T-cell subsets

**DOI:** 10.3389/fimmu.2025.1528707

**Published:** 2025-01-30

**Authors:** Chun-Mei Wang, Ying-Jie Chen, Bo-Cheng Yang, Jia-Wen Yang, Wei Wang, Yang Zeng, Jun Jiang

**Affiliations:** ^1^ Department of General Surgery (Thyroid Surgery), Affiliated Hospital of Southwest Medical University, Luzhou, Sichuan, China; ^2^ Department of Thyroid, Head, Neck and Maxillofacial Surgery, Mianyang Third People’s Hospital, Mianyang, Sichuan, China; ^3^ Department of Orthodontics, Affiliated Stomatology Hospital of Southwest Medical University, Luzhou, Sichuan, China

**Keywords:** experimental autoimmune thyroiditis, vitamin D, lymphocytes, differentiation, T-cell

## Abstract

**Objective:**

People with Hashimoto’s thyroiditis (HT) often have low vitamin D3 concentrations. Some research has suggested that vitamin D3 supplementation reduces thyroid inflammation, but this remains controversial.

**Methods:**

EAT was induced in female NOD/ShiLtJ mice by giving them water containing 0.05% sodium iodide, and 1μg/kg of 1α,25-(OH)_2_D_3_ was injected intraperitoneally every other day. After 8 weeks, the morphological architecture of the mouse thyroid follicles was examined by histological sections, thyroid autoantibodies and thyroid hormone concentrations were determined by enzyme-linked immunosorbent assays (ELISAs), and the major functions and subsets of B- and T-lymphocytes in the mouse thyroid were determined by tissue multiple immunofluorescence technology and ELISA.

**Results:**

EAT caused thyroiditis follicle destruction and interfollicular lymphocyte infiltration in mice, increased concentrations of circulating thyroid autoimmune antibodies TG-Ab and TPO-Ab, and abnormal thyroid hormone levels. EAT also increased the number and functionality of CD4+ Tfh, Th17,Th1 and Th2 cells in the thyroid, while decreasing the number and functionality of CD4+ Treg cells and CD19^+^B10 cells. Treatment with VD3 reversed these changes.

**Conclusion:**

Vitamin D3 supplementation can effectively treat autoimmune thyroiditis in mice. VD3 reduces autoimmune thyroid damage and decreases serum thyroid antibody levels in mice by inhibiting the differentiation and functionality of pro-inflammatory Tfh, Th17, Th1 and Th2 cells and by facilitating the differentiation and functionality of anti-inflammatory B10 cells and Treg.

## Introduction

Hashimoto’s thyroiditis (HT) is an organ-specific autoimmune disorder resulting from a mixture of hereditary factors and environmental triggers, characterized by lymphocytes infiltration of the thyroid gland and the production of autoantibodies against thyroid tissue (1). It is also the etiology of hypothyroidism in regions where iodine levels are adequate (2). Both T and B lymphocytes play a significant role in the pathogenesis of HT (3, 4). During the progression of HT, CD4+ T lymphocytes infiltrate and gradually replace normal thyroid follicular epithelial cells, leading to fibrosis and sclerosis of thyroid tissue and ultimately to hypothyroidism (5). Th2 and Th1 cells are critical in the pathogenesis of autoimmune thyroiditis. Th1 initiates the autoimmune process via cytokines and chemokines (6), and inflammation can be exacerbated by an imbalance of Th1/Th2 cells (7). In addition, recent research has demonstrated that the involvement of other B and T lymphocytes in the pathogenesis of autoimmune thyroiditis. An alteration in the ratio of Th17/Treg cells, together with an increasing in the population of Tfh cells, may be involved in the progression of HT (8, 9). Tfh cells play a critical role in maintaining humoral immunity by facilitating B cell differentiation, germinal center formation, and antibody production by memory B cells (10). Regulatory B cells that secrete IL-10 are called B10 cells and have the functionality to inhibit inflammatory damage in autoimmune diseases (11). Their reduction in number or functional defects may be one of the causes of HT (12). B-lymphocyte activating factor (BAFF) is involved in the proliferation, survival, and antibody production of B lymphocytes (13). It may also be associated with the occurrence of HT, as its expression is increased in the thyroid tissue of HT individuals (14).

Vitamin D is a steroid hormone that is essential for maintaining bone health and calcium-phosphorus balance. Its activated form is 1,25-dihydroxyvitamin D3 (15). Vitamin D has immunoregulatory properties and may influence the development of autoimmune diseases by modulating both innate and acquired immunity (16). Low vitamin D levels are often associated with autoimmune diseases. There is evidence that autoimmune thyroid diseases, including HT, are linked to vitamin D insufficiency. However, the causal relationship between HT and vitamin D insufficiency is unclear (17). Patients with HT had lower serum vitamin D levels compared with healthy individuals, and there was an inverse relationship between TSH levels and vitamin D concentrations (18–21). Vitamin D supplementation can reduce serum concentrations of pro-inflammatory factors such as TNF-alpha and IFN-gamma, and thyroid autoantibodies in patients with HT (22), and regulate T-cell activity (23) and subset ratios (24–26) such as the Th17/Treg cell ratio.

However, the effect of vitamin D supplementation in the intervention of autoimmune thyroiditis has not been fully determined. Some researchers believe that vitamin D has no significant relationship with the onset or progression of HT (27, 28). There are some clinical intervention studies with unsatisfactory results (29). The regulation of lymphocyte differentiation by vitamin D in autoimmune thyroiditis is still insufficiently elucidated. In this research, the EAT model was reproduced using iodine-rich water (30, 31), and then the experimental animals were supplemented with 1α,25-dihydroxyvitamin D3. The effects of vitamin D supplementation on the levels of autoimmune thyroid antibodies, thyroid function, and differentiation and functionality of thyroid B- and T lymphocyte subsets were observed in the mouse.

## Materials and methods

### Animals and grouping

The animal experiment was approved by the Experimental Animal Center of Southwest Medical University. All procedures were performed in accordance with the Guide for the Care and Use of Experimental Animals (National Health, USA, 1996) and the Rules on the Administration of Experimental Animals of China (Ministry of Health, China, 2001).

Forty-eight 28-day-old female NOD/ShiLtJ mice (GemPharmatech, Jiangsu, China) were randomly divided into three groups (n=16/group): control, experimental autoimmune thyroiditis (EAT), and vitamin D3 supplementation (EAT+VD3). All three experimental groups were maintained in a Specific Pathogen-Free (SPF) environment that maintained appropriate temperature and humidity levels, controlled artificial circadian rhythms, and provided the mice with unrestricted access to a sawtooth maintenance chow and water. After one week of adaptive feeding, the 8-week experiment began. Controls received purified sterile drinking water, while the EAT group and the EAT+VD3 group received purified sterile drinking water supplemented with 0.05% sodium iodide(0.64 gNaI/L). 1α,25-(OH)2D_3_ (Sigma-Aldrich, CAS:32222-06-3) was first solubilized in 95% ethanol to prepare a 0.01 µg/10 µl concentration storage solution, and then the working solution was prepared according to the ratio of water: propylene glycol: ethanol ratio of 5:4:1. The 1α,25-(OH)2D_3_ solution was injected intraperitoneally into the EAT+VD3 group of mice at a dose of 1 µg/kg every other day (32). At the same time, the EAT mice were injected with propylene glycol and ethanol solutions without VD3.

**Figure 1:**
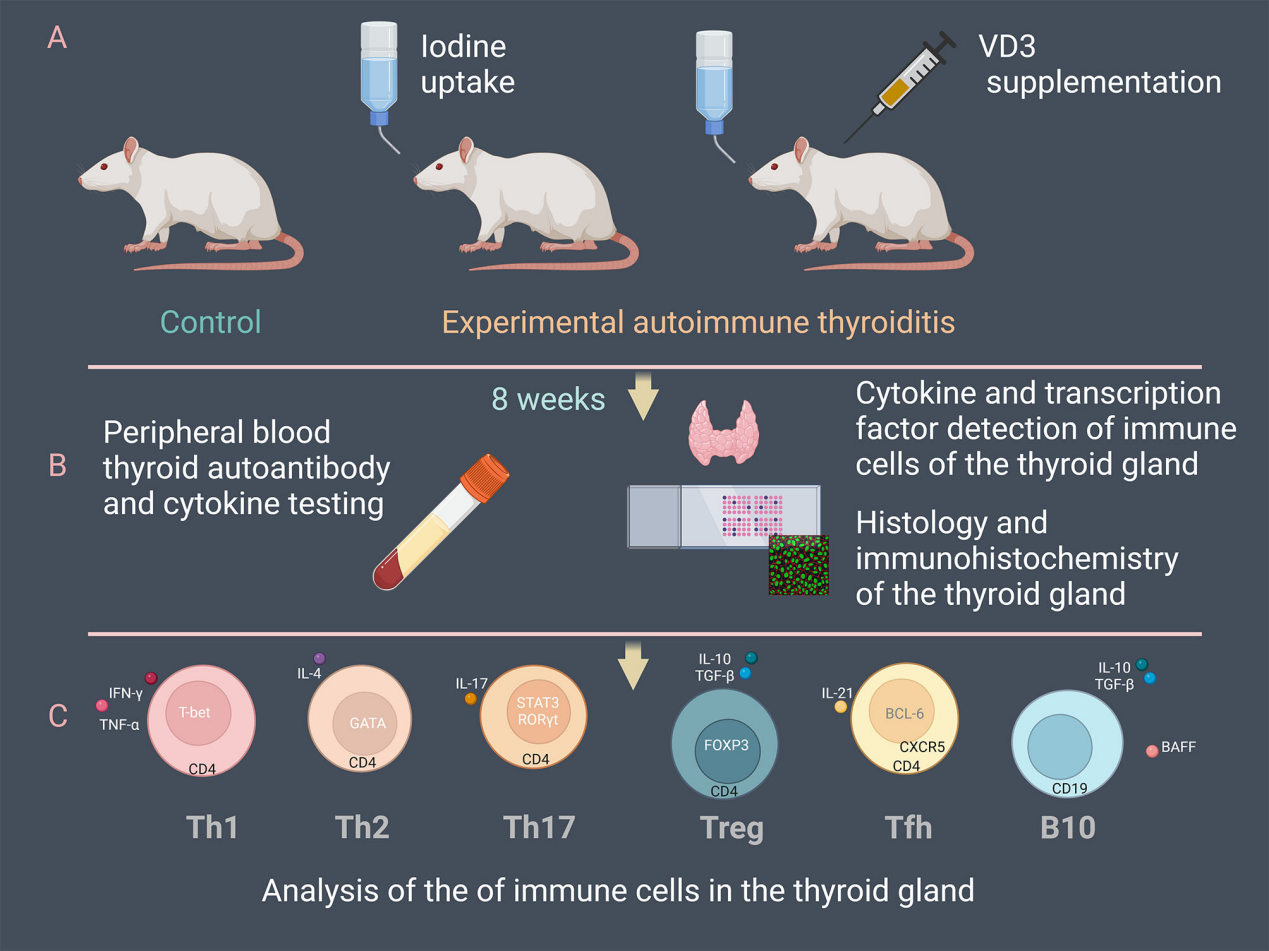
Overview of the experimental procedure. **(A)** 48 NOD/ShiLtJ mice were divided into three groups: control, experimental autoimmune thyroiditis (EAT) induced by drinking water supplemented with 0.05% iodine, and the treatment group (EAT+VD3) based on EAT. The EAT+VD3 group was injected intraperitoneally with 1α,25-(OH)2D_3_ at a dose of 1µg/kg every other day. **(B)** Mouse thyroid tissue and peripheral blood were collected at the end of eight weeks. Concentrations of thyroid autoantibodies, thyroid hormones and cytokines in peripheral blood were measured. Histologic sections were used to evaluate thyroid inflammation. Thyroid cytokines and transcription factors were determined by ELISA and immunohistochemistry. **(C)** Differentiation of Tfh, Th17, Treg, Th1, Th2 and B10 cells in the thyroid tissues was examined. The changes of the immune cells under the EAT environment and the effects of VD3 supplementation on their differentiation and functionality were evaluated.

At the end of 8 weeks, all mice were euthanized and thyroid tissue and peripheral blood were collected. Peripheral blood samples were kept at room temperature for 40 minutes and centrifuged at 4°C for 30 minutes at 3000 rpm. The supernatant serum was gently extracted and stored at -80°C for analysis by ELISA. Bilateral thyroid glands were removed from the mice for subsequent histologic and immunohistochemical examination (**Figure 1**).

### Detection of VD3, thyroid hormones and thyroid autoantibodies in serum

ELISA was used to detect 25-(OH)-VD_3_, TPO-ab, TG-ab, TT4, TT3, FT4, TSH and in mouse serum. The kits were purchased from FANKEL Industrial Co., Ltd, Shanghai, China. The assay was performed according to the manufacturer’s instructions for use.

### Determination of serum inflammatory factors and BAFF

TGF-beta, IL-4, IFN-gamma, IL-21, IL-10, TNF-alpha, IL-17and BAFF in mouse serum were determined by ELISA. The kits were purchased from FANKEL Industrial Co., Ltd, Shanghai, China. The assay was performed according to the manufacturer’s instructions for use.

### Thyroid tissue pathology

Mouse thyroid tissue samples were preserved in 4% paraformaldehyde, followed by paraffin embedding. The samples were sectioned at 4 μm and stained with hematoxylin and eosin. Two investigators separately observed the thyroid follicular structure and assessed the extent of lymphocyte infiltration using a light microscope. They then graded the extent of thyroid lymphocyte infiltration based on the quantification of lymphocytes present. The grading standard was: no lymphocyte infiltration - score 0, the infiltration area was less than 10% - score 1, the infiltration area was 10-20% - score 2, the infiltration area was 20-50% - score 3, and the lymphocyte infiltration range was more than 50% - score 4 (33).

### Detection of the expression of transcription factors and cytokines of T cells and B cells

IFN-γ, TNF-α, T-bet, IL-4, GATA3, IL-17, ROR γ T, STAT3, FOXP3, IL-21, Bcl-6, CXCR5, BAFF, IL-10 and TGF-β in mouse thyroid tissue were detected by enzyme-linked immunosorbent assay (ELISA) kit. ELISA kits were purchased from FANKEL Industrial Co., Ltd, Shanghai, China. The assay process was performed according to the manufacturer’s instructions.

### Tissue multiple immunofluorescence staining

Paraffin-embedded thyroid tissue samples were first deparaffinized and dehydrated, followed by antigen retrieval with hydrogen peroxide and serum. Using the TSA technique, anti-CD4 antibody (Servicebio, Wuhan, China), anti-CD19 antibody (Servicebio, China), anti-RORγT antibody (Aibixin, Shanghai, China), anti-CXCR5 antibody (R&D Systems, USA), anti-IFN-gamma antibody (R&D Systems, USA), anti-IL-10 antibody (Servicebio, Wuhan, China), anti-FOXP3 antibody (Servicebio, Wuhan, China), anti-BAFF antibody (R&D Systems, USA), and anti-IL-4 antibody (Novus, USA) were incubated with the tissue sections overnight at 4°C in a humidified chamber. After eluting the unbound primary antibody, the fluorescent secondary antibody (Servicebio, Wuhan, China) was incubated with the sections in a humidified chamber at four degrees Celsius for fifty minutes in the dark. Finally, the nuclei were labeled with DAPI (Servicebio, Wuhan, China) and the sections were mounted in mounting medium. The fluorescence signals of the thyroid tissue sections were examined under an upright fluorescence microscope (Nikon Eclipse C1) and photographed for analysis.

### Statistical analysis

Data analysis and graphs were GraphPad Prism version 8.0. All results are presented as means with standard deviation (SD) or as percentages. Comparative analyses between groups were performed using one-way analysis of variance (ANOVA). A significance level of 95% confidence interval was used. Statistical significance of p-values is indicated as follows: ns: not significant; *: P<0.05; **: P<0.01; ***: P<0.001; ****: P<0.0001.

## Results

### Vitamin D3 treatment alleviates experimental autoimmune thyroiditis in mice

Histopathologic examination revealed that all mice in this experiment developed experimental autoimmune thyroiditis (EAT) after drinking iodine-containing water for 8 weeks. The extent of thyroiditis was assessed by hematoxylin-eosin staining of paraffin-embedded thyroid gland sections and by measuring the concentrations of thyroid autoantibodies in the serum. In the EAT+VD3 group, EAT mice were supplemented with active vitamin D3 (1,25 dihydroxy vitamin D3) by intraperitoneal injection for 8 weeks. Monitoring of the experimental animals indicated that vitamin D3 supplementation did not have a significant impact on their body weight. (**Supplementary Figure 1**).

**Figure 2:**
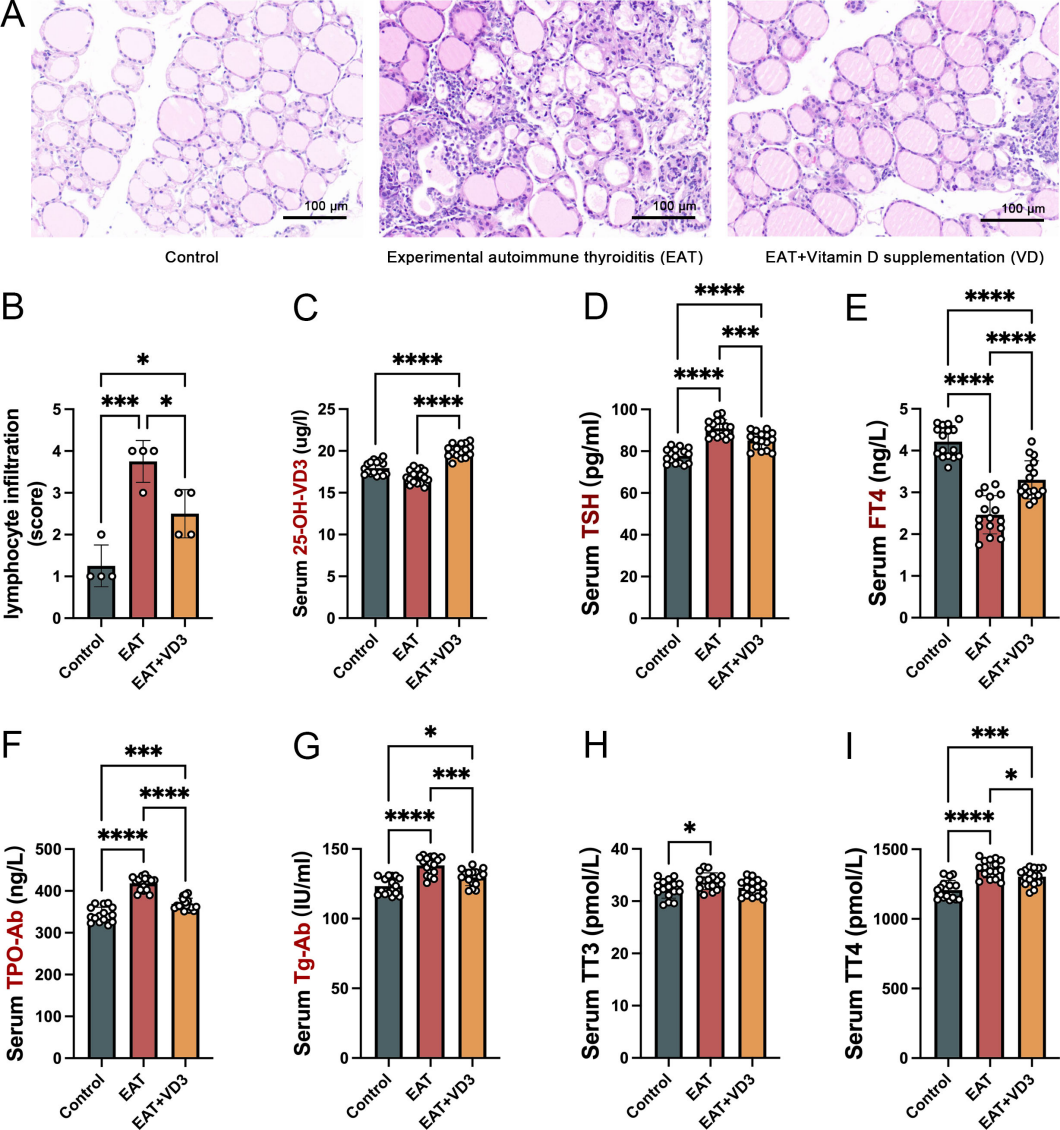
VD3 alleviated thyroid follicle destruction and lymphocyte infiltration caused by autoimmune thyroiditis in mice and reduced thyroid autoantibody levels. **(A)** Iodine-containing drinking water caused damage to the structure of thyroid follicles in mice, with large numbers of lymphocytes infiltrating between the follicles. Vitamin D3 supplementation reduced follicular cell destruction and lymphocyte infiltration. n=4 **(B)** Relative quantitative analysis of infiltrating lymphocytes in thyroid tissue showed that VD3 reduced the rate of lymphocyte infiltration caused by EAT. n=4 **(C)** Serological tests confirmed that intraperitoneal injection of active VD3 upregulated serum VD3 levels in mice. n=16 **(D, E)**. In comparison to the control group, the serum levels of TSH in the EAT mice were found to be elevated, whereas the serum levels of FT4 were diminished. Notably, the administration of vitamin D3 was able to partially mitigate these alterations. n=16 **(F, G)**. Mice drinking iodine-containing water had elevated levels of peripheral blood thyroid autoantibodies TG-Ab and TPO-Ab, confirming the development of autoimmune thyroiditis. Supplementation with VD3 reduced the levels of thyroid autoantibodies. n=16 **(H, I)**. The levels of thyroid hormones in mice consuming iodine-containing water changed, characterized by abnormal concentrations of TT3 and TT4 in peripheral blood. Furthermore, the study found that supplementation with vitamin D3 could partially restore the observed deviation in TT4 levels. n=16 Results are presented as mean ± SD. *, P<0.05; ***, P<0.001; ****, P<0.0001.

Compared with the control group (normal mouse thyroid), the thyroid follicles in the EAT mouse were partially destroyed and incomplete (**Figure 2A**), and the lymphocyte infiltration rate around the follicles was significantly increased (**Figure 2B**), whereas VD3 supplementation alleviated the thyroid follicular cell damage and lymphocyte infiltration. Intraperitoneal injection of active VD3 upregulated serum VD3 levels in mice (**Figure 2C**). In EAT mice, there were significantly higher levels of serum thyroid autoantibodies TG-Ab and TPO-Ab, along with abnormal levels of TSH, FT4, TT4 and TT3 (**Figures 2D, E, H, I**). Whereas VD3 supplementation reduced the elevated levels of TG-Ab and TPO-Ab (**Figures 2F, G**) and partially reversed the abnormal TSH, FT4 and TT4 levels (**Figures 2D, E, I**).

### Vitamin D3 reduces serum pro-inflammatory factors and upregulates anti-inflammatory factors in EAT mice

**Figure 3:**
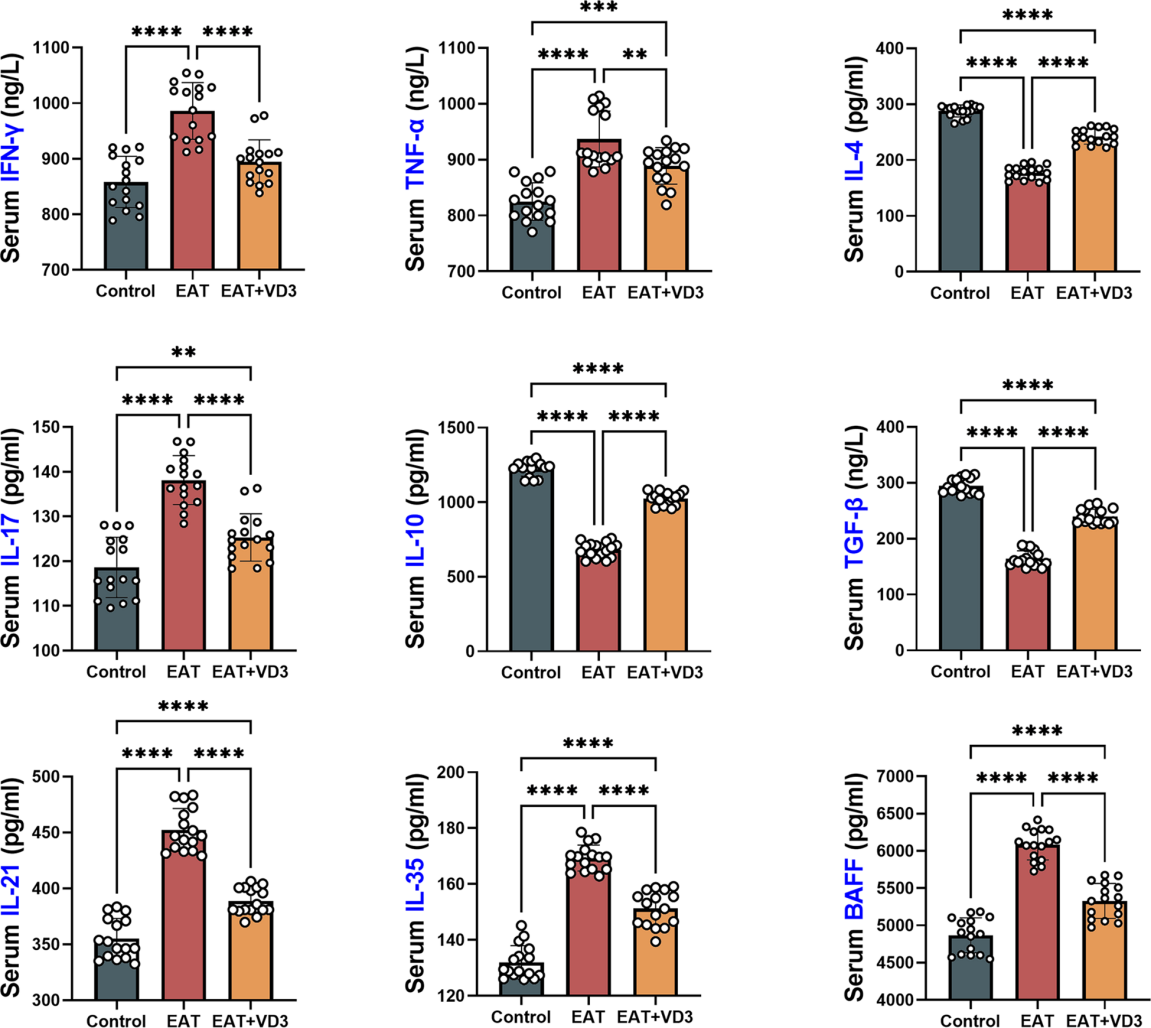
Effects of VD3 supplementation on serum inflammatory and anti-inflammatory factors in EAT mice. Compared to the control group, serum levels of the pro-inflammatory factors IFN-gamma, IL-21, IL-17, TNF-alpha, and BAFF were increased in EAT mice, while the levels of the anti-inflammatory factors IL-10, TGF-beta, and IL-4 were decreased. VD3 supplementation could reverse the changes in the levels of these inflammatory factors. n=16. Results are presented as mean ± SD. **, P<0.01; ***, P<0.001; ****, P<0.0001.

Mouse serum concentrations of IL-10, TGF-beta, IL-21, IL-4, IFN-gamma, TNF-alpha, IL-17 and BAFF were measured by ELISA. Compared with the control group, serum concentrations of the pro-inflammatory factors IFN-gamma, IL-21, IL-17, TNF-alpha, and BAFF were increased in EAT mice, whereas the concentrations of the anti-inflammatory factors IL-10, TGF-beta, and IL-4 were decreased. Supplementation with active vitamin D3 can downregulate the levels of IL-21, TNF-alpha, IL-17, IFN-gamma, and BAFF in EAT mice, while upregulating the levels of IL-10, TGF-beta, and IL-4 (**Figure 3** **).**

### Vitamin D3 regulates Th1/Th2 cell balance by affecting the functionality and number of Th2 and Th1 cells

In this study, the number of Th1 and Th2 cells in mouse thyroid sections was determined by multiplex immunofluorescence. IFN-gamma-positive staining in thyroid tissue sections was defined as Th1 cells, and IL-4-positive staining was defined as Th2 cells. GATA3 and T-bet serve as transcription factors for Th2 and Th1 cells, respectively (34, 35).

**Figure 4:**
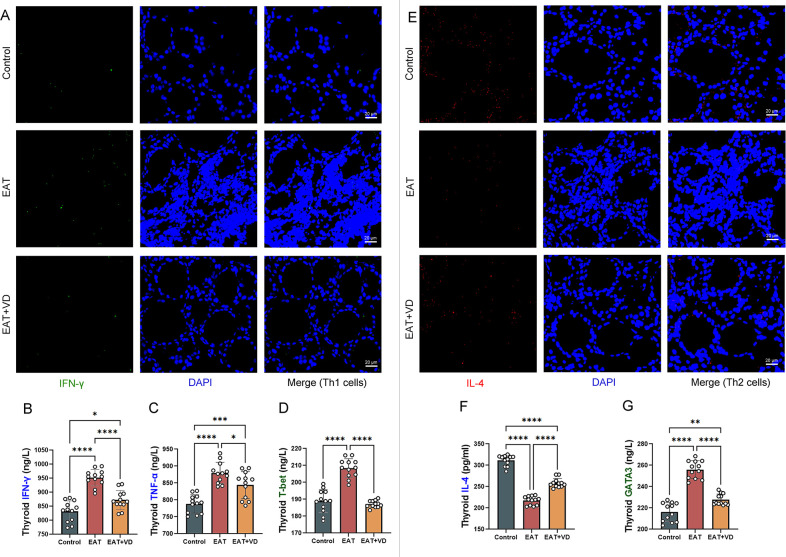
VD3 supplementation regulated the number of Th2 and Th1 cells in the thyroid and the levels of associated cytokines and transcription factors in EAT mice. **(A)** IFN-γ positive staining in tissue immunofluorescence are Th1 cells. The amount of Th1 cells in the thyroid gland of EAT mice was observed to be higher than that in control mice, and the administration of VD3 supplementation showed a partial ameliorative effect on this elevation. n=4 **(B-D)** T-bet, TNF-alpha, and IFN-gamma were elevated in the thyroid tissue of the EAT mice. The expression levels of these cytokines and transcription factors can be reduced by VD3 supplementation. That is, VD3 down-regulated the number and functionality of Th1 cells in the thyroid gland of EAT mice. n=12 **(E)** IL-4- positive staining under tissue immunofluorescence are Th2 cells. The amount of Th2 cells in the thyroid tissue of EAT mice was decreased, while VD3 supplementation could partially reverse the decrease in the amount of Th2 cells. n=4 **(F, G)** The expression of IL-4 was decreased in the thyroid tissue of EAT mice, while the expression of the transcription factor GATA3 was increased. VD3 supplementation increased IL-4 levels and decreased GATA3 expression. That is, VD3 upregulates the number and activity of Th2 cells in the thyroid gland of EAT mice. n=12 Results are presented as mean ± SD. *, P<0.05; **, P<0.01; ***, P<0.001; ****, P<0.0001.

Compared with the control group of mice, the thyroid tissue of EAT mice showed an increase presence of Th1 cells, however, administration of active vitamin D3 resulted in a reduction of Th1 cell populations in the EAT mice (**Figure 4A**). Enzyme-linked immunosorbent assay showed that EAT resulted in an increased secretion of the cytokines TNF-alpha and IFN-gamma, along with an upregulation of the transcription factor T-bet in mouse thyroid tissue, whereas VD3 reversed these abnormalities (**Figures 4B–D**).

On the other hand, the number of Th2 cells in the thyroid tissue of EAT mice was reduced, whereas treatment with VD3 increased the number of Th2 cells (**Figure 4E**). There was a downregulation of the cytokine IL-4 in the thyroid tissue of EAT mice, whereas VD3 could upregulate its levels. (**Figure 4F**). Increased expression of the transcription factor GATA was observed in the thyroid tissue of mice with EAT. VD3 reversed this abnormality. (**Figure 4G**).

These results suggest that VD3 ameliorates EAT-induced Th1/Th2 cell numbers and functional abnormalities by affecting transcription factor expression and cytokine secretion.

### Vitamin D3 promotes Treg cell differentiation and functionality and inhibits Th17 cell differentiation and functionality

**Figure 5:**
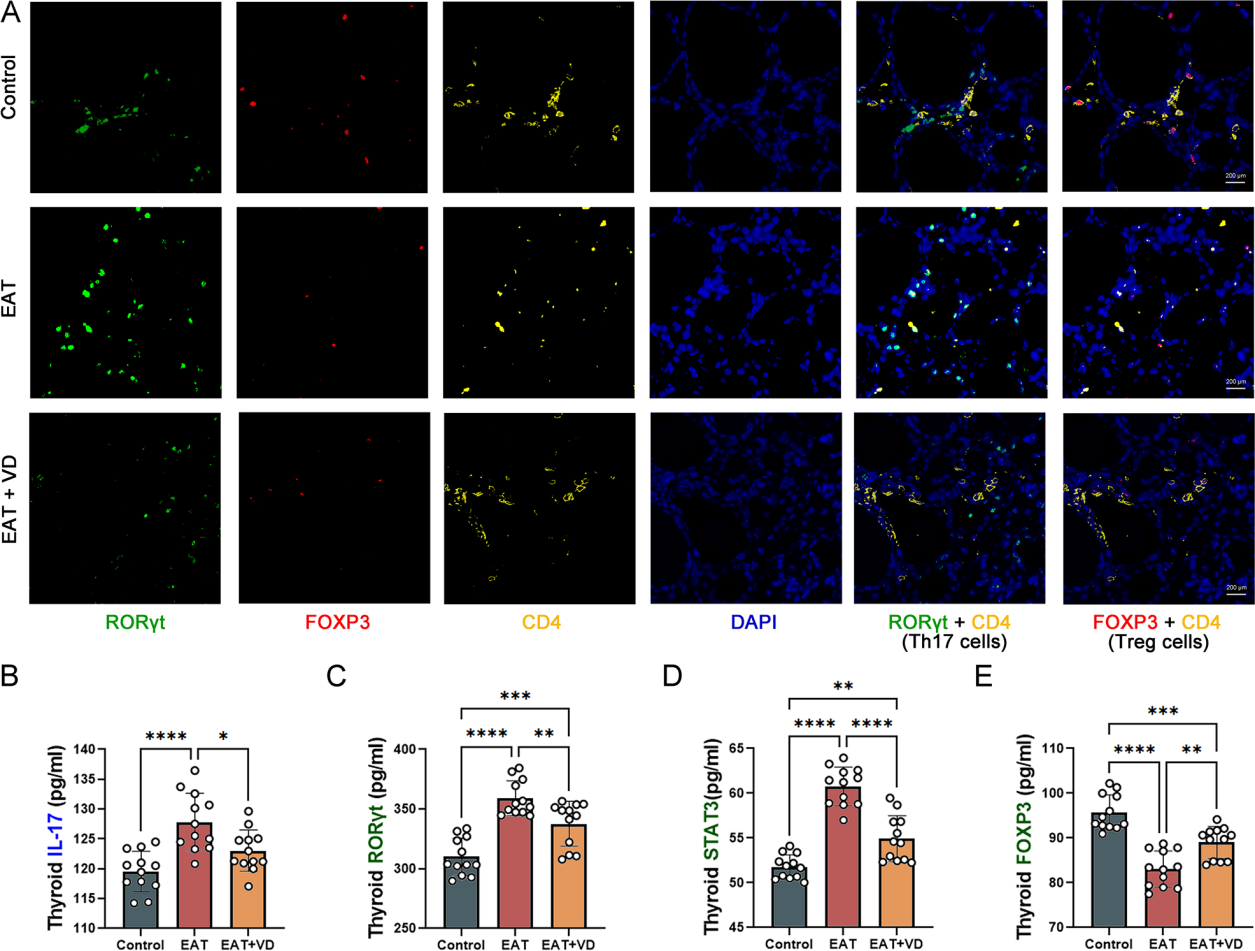
EAT decreased the number and functionality of Treg cells and increased the number and functionality of Th17 cells in the mouse thyroid, and VD3 reversed these changes. **(A)** In multiple immunofluorescence staining of the mouse thyroid gland, cells that expressed both FOXP3 and CD4 were defined as Treg cells, and cells expressing both RORγT and CD4 were defined as Th17 cells. In EAT mice, there was a decrease in Treg cells and an increase in Th17 cells compared to the control group. While treatment with VD3 resulted in an increase in Treg cells and a decrease in Th17 cells. n=4 **(B-D)** The thyroid gland in EAT mice produced higher amounts of IL-17, and the expression of transcription factors STAT3 and RORγT was increased.VD3 treatment decreased the production of IL-17 and the expression of STAT3 and RORγT. n=12 **(E)** In EAT mice, the levels of the transcription factor FOXP3 were decreased, whereas treatment with VD3 increased its expression. n=12 Results are presented as mean ± SD. *, P<0.05; **, P<0.01; ***, P<0.001; ****, P<0.0001.

In this study, multiple immunofluorescence techniques were used to identify Treg and Th17 in mouse thyroid tissue. Cells expressing both RORγT and CD4 were defined as Th17 cells (36), and cells expressing both FOXP3 and CD4 were defined as Treg cells (37). The results showed that there was a decrease in Treg cells and an increase in Th17 cells in the thyroid tissue of EAT mice. However, treatment with VD3 resulted in a decrease in Th17 cells and an increase in Treg cells (**Figure 5A**). EAT resulted in an increase in IL-17, a cytokine produced by Th17 cells (**Figure 5B**), and the expression of RORγT and STAT3, two transcription factors of Th17 cells, were also increased (**Figures 5C, D**), while the expression of the transcription factor FOXP3 in Treg cells was decreased (**Figure 5E**), and VD3 treatment reversed these changes. These results indicate that VD3 ameliorates the number and functional abnormalities of Treg and Th17 cells caused by EAT by affecting transcription factor expression and cytokine secretion.

### Vitamin D3 down-regulated EAT-induced increase in number and functionality of Tfh cells

**Figure 6:**
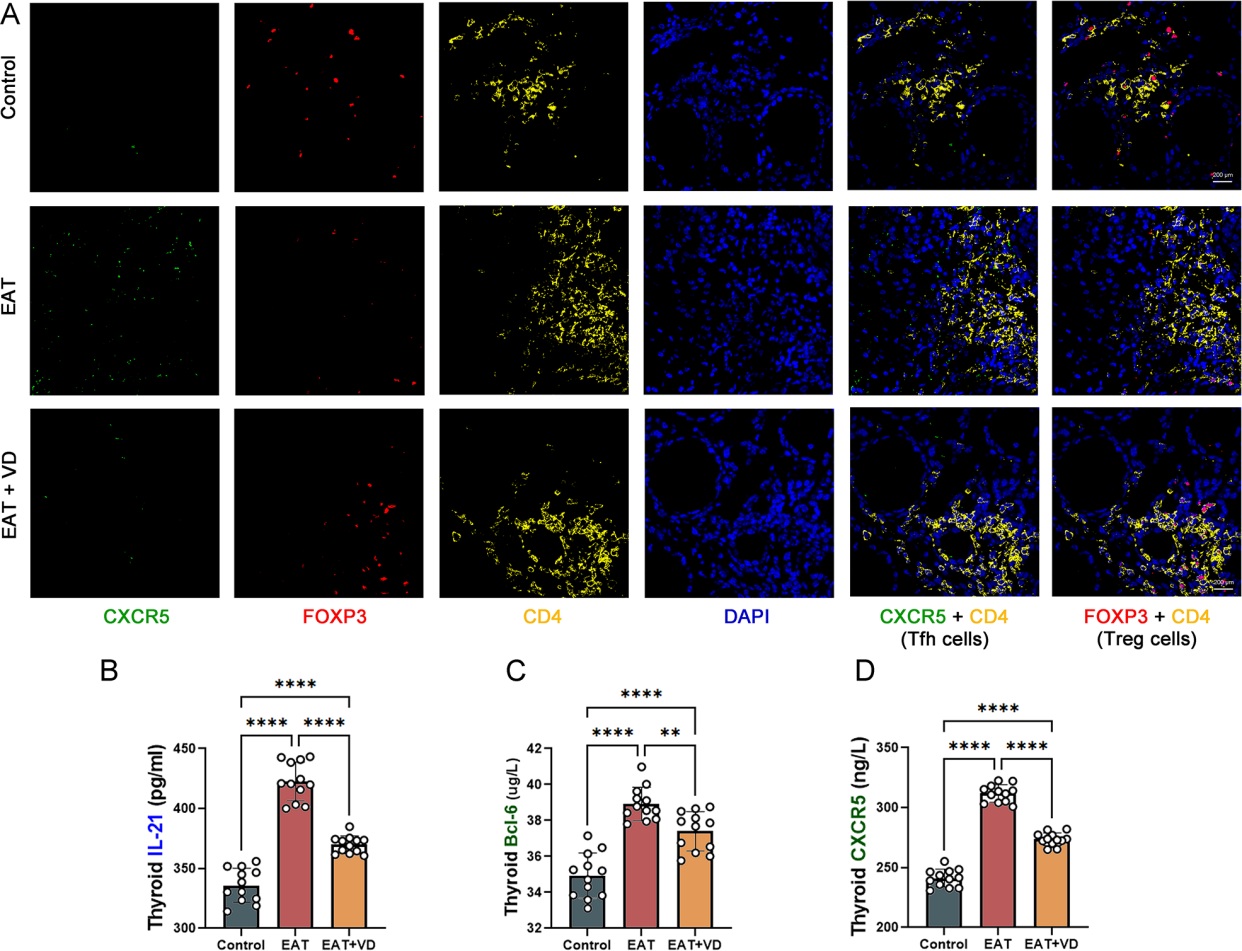
EAT increases the number and function of Tfh cells in the mouse thyroid, whereas VD3 downregulates the number and function of Tfh cells. **(A)** Cells staining positive for both CXCR5 and CD4 are Tfh cells, and cells staining positive for both FOXP3 and CD4 are Treg cells. Compared to the control group, Tfh cell levels increased, while Treg cell levels decreased in the thyroid gland of EAT mice. Supplementation with VD3 can partially increase the number of Treg cells and decrease the number of Tfh cells. n=4 **(B-D)** EAT resulted in increased levels of the Tfh cell-associated cytokine IL-21, increased expression of the surface receptor CXCR5, and increased levels of the transcription factor BCL-6 in the thyroid tissue of mice. VD3 treatment reversed the upregulation of IL-21, BCL-6 and CXCR5. n=12 Results are presented as mean ± SD.**, P<0.01; ****, P<0.0001.

The number of Treg cells and Tfh cells in the mouse thyroid was determined by multiple immunofluorescence. Cells positive for both CD4 and CXCR5 were defined as Tfh cells (37). The results indicated that EAT caused an increase in the number of Tfh cells in mouse thyroid, while VD3 treatment downregulated their number (**Figure 6A**). Compared to the normal mouse thyroid, the concentration of IL-21 secreted by Tfh was increased in the thyroid of EAT mice (**Figure 6B**). In addition, there was an increase in the expression of the transcription factor Bcl-6 (**Figure 6C**), and an upregulation of the cell surface receptor CXCR5 (**Figure 6D**). Treatment with VD3 reversed these changes. These results indicate that EAT causes abnormalities in both the number and functionality of Tfh cells, and that VD3 reverses these changes by affecting the cytokine secretion and transcription factor expression characteristic of Tfh cells.

### Vitamin D3 upregulated the number and function of B10 cells and reduced BAFF levels in the thyroid of EAT mice

**Figure 7:**
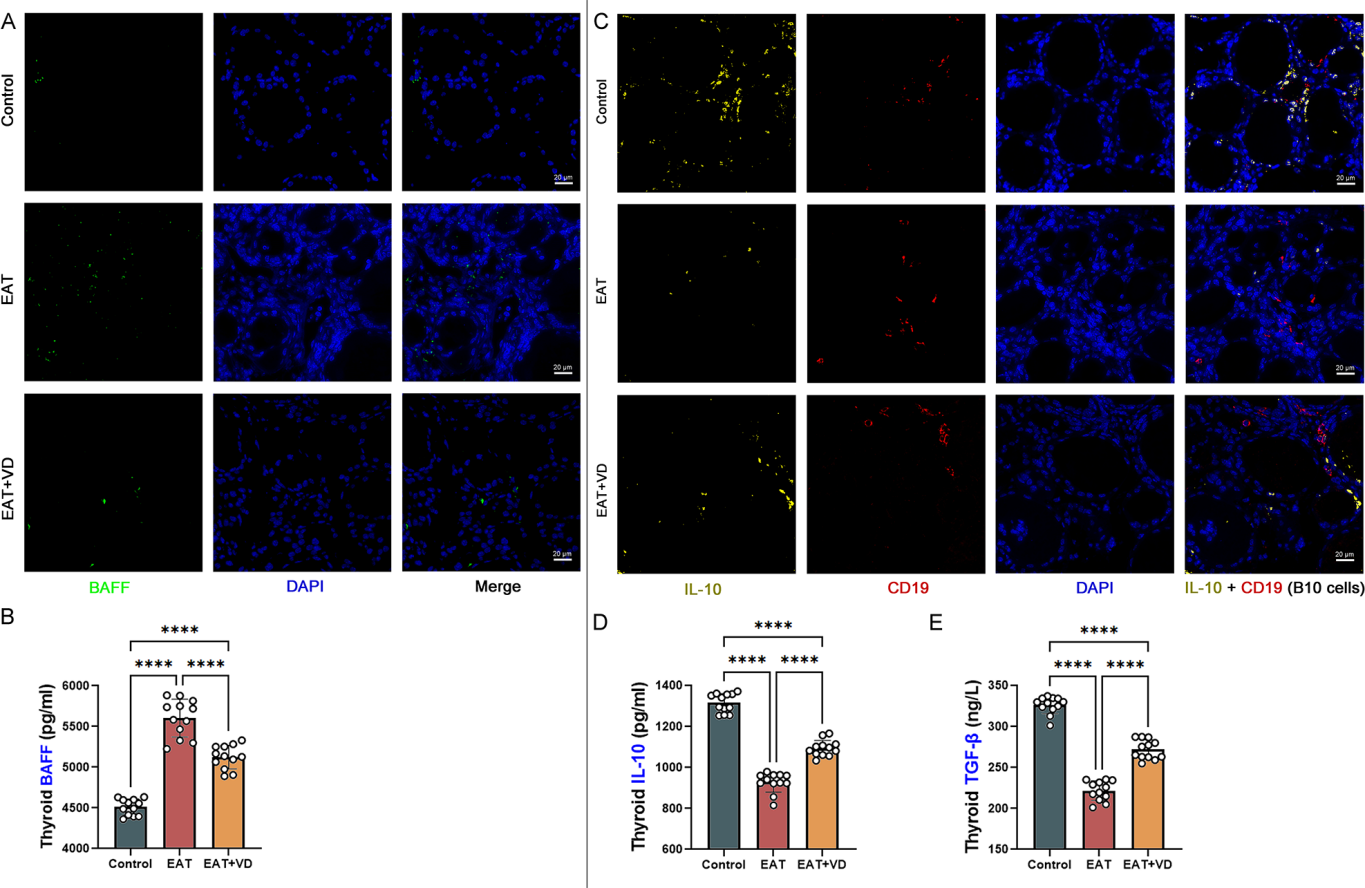
VD3 downregulates the level of BAFF and upregulates the amount of regulatory B cells, B10 cells. **(A)** The primary role of BAFF is to maintain the survival of B cells and promote their development into plasma cells that produce antibodies. Immunofluorescence showed that EAT led to upregulation of BAFF levels in the mouse thyroid, while VD3 downregulated BAFF. n=4 **(B)** Quantitative detection of BAFF by enzyme-linked immunosorbent assay also showed that EAT upregulated BAFF while VD3 downregulated BAFF. n=12 **(C)** In this study, cells that were CD19 positive and produced IL-10 were defined as B10 cells. Multiple immunofluorescence analyses indicated that EAT resulted in a reduction of B10 cell populations within the murine thyroid. n=4 **(D, E)** B10 cell-related cytokines TGF-beta and IL-10 were reduced by EAT, whereas VD3 treatment reversed the changes in TGF-beta and IL-10. n=12 Results are expressed as mean ± SD. ****, P<0.0001.

B cell activating factor (BAFF) is so named because it can stimulate the growth of B cells. It is produced by myeloid cells such as macrophages, neutrophils, dendritic cells, and monocytes. Its primary function is to maintain the survival of B cells and promote their development into plasma cells, which are responsible for antibody production. A specific subset of regulatory B cells, known as B10 cells, plays a critical role in modulating of the immune response by exerting a suppressive effect. Immunofluorescence (**Figure 7A**) and enzyme-linked immunosorbent assay (**Figure 7B**) showed that EAT led to upregulation of BAFF levels in mouse thyroid tissue, whereas VD3 treatment could downregulate BAFF levels. In multiple immunofluorescence of mouse thyroid sections, cells staining positive for both CD19 and IL-10 were B10 cells (38, 39). EAT caused a reduction in the number of B10 cells in the mouse thyroid (**Figure 7C**), as well as a reduction in the levels of the B10-related cytokines IL-10 and TGF-beta (**Figures 7D, E**). Supplementation with VD3 can increase the amount of B10 cells and improve the concentrations of TGF-beta and IL-10. The results suggest that VD3 alleviates EAT-induced thyroid inflammation by decreasing BAFF levels and increasing the number and function of regulatory B10 cells.

## Discussion

Vitamin D is significant in the modulation of autoimmune diseases (40). Vitamin D deficiency is prevalent among individuals diagnosed with HT (41), and the concentration of vitamin D exhibits an inverse relationship with the risk of HT (42). Some studies have shown that supplementation with VD may help alleviate thyroiditis (18), but it remains controversial (43). At present, the relationship between VD and autoimmune thyroid disease remains inadequately elucidated, and the benefit of VD supplementation in this disease remains questionable (22). For a long time, some clinicians have prescribed vitamin D3 to patients with Hashimoto’s thyroiditis in an effort to treat or alleviate the condition, although this practice remains a topic of debate. This research investigated the effects of VD3 on EAT-induced thyroid histology, serum thyroid autoantibodies, and thyroid hormone concentrations. Notably, we found that serum TSH, TT4, and TT3 levels were higher in the EAT group compared to the control group, and we also found that their serum FT4 levels were reduced. Hashitoxicosis, a transient hyperthyroidism caused by inflammation associated with Hashimoto’s thyroiditis, was first described by Fatourechi in 1971 (44). Previous studies have shown that serum TSH concentration is increased in EAT mice (45), while serum TT4 concentration increases some time after the start of modeling (35-60 days) and is positively correlated with changes in serum TG-ab concentration (46). Studies in rats have shown that EAT results in a significant increase in serum TT3, but a significant decrease in FT4 (47).Therefore, we believe that the increase in TSH is mainly due to the decrease in FT4, while the increase in total T3/T4 is thought to be due to the release of thyroid hormone into the circulation after the destruction of the thyroid follicles. The results showed that VD3 improved EAT-induced thyroid follicular destruction and lymphocyte infiltration, reduced serum thyroid autoantibody levels, and partially reversed abnormal thyroid hormone levels.

We investigated the effect of vitamin D3 on both the quantity and functionality of intrathyroidal lymphocytes induced by EAT. Research indicates that the production of cytokines is a critical function of activated T cells (48). Based on the characteristic cytokines and transcription factors, we assessed both the quantity and functionality of the CD4+ T cell subsets, focusing specifically on Tfh, Th17, Th2, Th1, and Treg cells. Tfh, Th17, Th2, and Th1 cells induce immune damage to the thyroid gland by releasing inflammatory mediators, whereas Treg cells exert immunosuppressive effects. The importance of alterations in the number and functionality of Th2 and Th1 cells and the imbalance between the two in autoimmune thyroiditis has been widely reported (7). The proportion of Th17/Treg cells is increased in individuals diagnosed with HT (49). Treg cells inhibit the differentiation and activity of Th17, Th1, and Th2 cells in AITD and help to preserve thyroid tissue (3). Tfh cells also play a pivotal role in autoimmune diseases (50). For example, Tfh cells are increased in the thyroid tissue of individuals diagnosed with HT (9, 51, 52). They are located within lymphoid follicles, and their main function is to support B cell proliferation and antibody production, and to participate in humoral immunity. Importantly, IL-21 produced by Tfh cells exerts an inhibitory effect on both the differentiation and functionality of Treg cells (53, 54). This experiment showed that EAT caused an increase in both the number and functionality of Tfh, Th17, Th2 and Th1 cells within the thyroid gland of mice, a decrease in the number and weakening of Treg cells, and VD3 supplementation reversed these phenomena.

B lymphocytes also play a pivotal part in autoimmune thyroiditis. BAFF is a key cytokine that plays an important role in regulating B cell survival, differentiation, and the antibody production. It is increased in HT (13, 14). In this study, EAT upregulated the level of BAFF in the mouse thyroid, while vitamin D3 treatment downregulated BAFF. Breg cells have the function of suppressing inflammatory responses. Their reduced number and functional defects affect the severity and recovery of autoimmune diseases. IL-10-producing Breg cells (B10 cells) are particularly important in the regulation of autoimmune diseases (55, 56). B10 cells suppress inflammation by regulating T cell differentiation (57–59). For example, B10 cells secrete immunomodulatory factors such as TGF-beta, induce Th1 cell apoptosis (60), and reduce CD8+ effector T cell responses (61). In this study, these phenomena were observed in thyroid tissue from EAT mice and VD3 at least partially reversed these abnormalities.

This study also has several limitations. First, compared with regular NOD mice, NOD.H2-h4 mice are susceptible to autoimmune thyroiditis but resistant to spontaneous diabetes (62, 63), which is more suitable for our experimental design. Unfortunately, we had to select NOD mice for the experiment due to the limited conditions during our experimental period. Previous studies have shown that female NOD mice do not develop diabetes at 15 weeks of age (64, 65), and the median time of diabetes onset is 19-20 weeks of age (65, 66). In our experimental design, we used 4-week-old female NOD mice to induce autoimmune thyroiditis by high iodine intake after 1 week of adaptive feeding. The experimental endpoint was within the 8th week of high iodine induction, i.e. within the 13th week of the mice’s life. We believe that this design reduces the confounding effect of spontaneous diabetes on the experiment. Second, vitamin D3 was administered at the same time as high-iodine induced EAT. If EAT was present and severe before vitamin D3 supplementation, further experiments are needed to investigate whether vitamin D3 supplementation is effective under such circumstances. Besides, given that autoimmune thyroiditis is a chronic condition, it is essential to conduct extensive experimental studies to assess the long-term impacts of vitamin D3 on EAT and the differentiation and function of intrathyroidal lymphocytes in the future. Our experiment has some shortcomings in certain detection methods and approaches. Although 25(OH) vitamin D3 is a circulating substance in human studies (67–71), there are also studies showing that after injecting 1,25(OH)2 vitamin D3, the trends of 25(OH) vitamin D3 and 1,25(OH)2 vitamin D3 in mice are the same (32). However, it is also necessary to maintain the consistency of the analytes. Our research work has been limited by certain experimental conditions, such as restrictions on the use of equipment at our institution. Therefore, when detecting the concentration of vitamin D3, we referred to some literature and chose the relatively simple ELISA as the detection method (72, 73). Despite these limitations, our work still provides a significant observational basis and mechanism discussion for exploring the effects of vitamin D3 on the course of autoimmune thyroiditis.

## Conclusion

**Figure 8:**
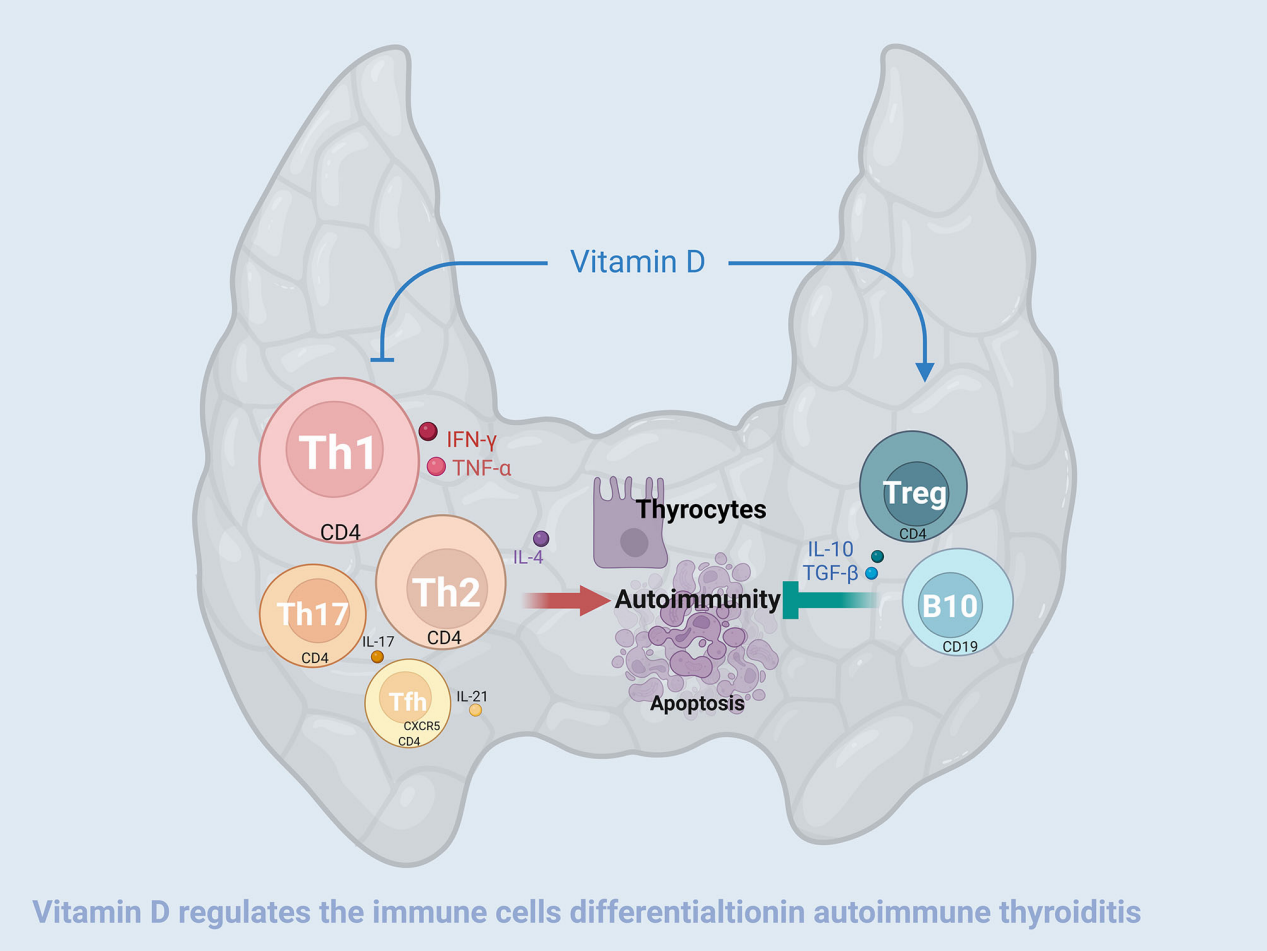
Vitamin D3 alleviates experimental autoimmune thyroiditis by regulating thyroid lymphocyte differentiation. High iodine-induced EAT is manifested by an increase in the number and enhanced function of CD4-positive Tfh, Th17, Th2, and Th1 cells, and a decrease in the number and functional activity of CD4-positive Treg cells and CD19-positive B10 cells that secrete IL-10. Treg and B10 cells have the function of inhibiting the differentiation and functionality of Tfh, Th17, Th2 and Th1 cells and are also negatively modulated by the former. On the one hand, vitamin D3 downregulates the increase in the quantity and functional activity of Tfh, Th17, Th2, and Th1 cells, and on the other hand, it upregulates the decrease in the quantity and functional activity of Treg and B10 cells, thereby alleviating the thyroid damage caused by EAT.

Experimental autoimmune thyroiditis in mice resulted in an increase in both the number and functional activity of CD4-positive Tfh, Th17, Th2, and Th1 cells, and a decrease in both in the number and function of CD19-positive B10 cells and CD4-positive Treg cells. The abnormalities in the function and numbers of these B and T lymphocytes were partially reversed by vitamin D3 supplementation (**Figure 8**). Correspondingly, the thyroid follicular damage and the increase in serum thyroid autoantibodies caused by EAT were also ameliorated by VD3 treatment. These lines of evidence demonstrated that VD3 supplementation is effective in treating experimental autoimmune thyroiditis in mice, and these improvements are closely related to the effects of VD3 on the differentiation and functionality of B and T lymphocytes.

## Data Availability

The original contributions presented in the study are included in the article/**Supplementary Material**. Further inquiries can be directed to the corresponding authors.

## Supplementary material

Supplementary Figure 1 Weight-age curves were documented for the control group, the EAT group, and the EAT+VD3 group. There was no significant difference among the three groups of experimental animals. n=16.
