# Supplementary figures and images for: Supplementation with active vitamin D3 ameliorates experimental autoimmune thyroiditis in mice by modulating the differentiation and functionality of intrathyroidal T-cell subsets
Source: Front Immunol. 2025 Jan 30;16:1528707. doi: 10.3389/fimmu.2025.1528707 (PMC11821646; doi:10.3389/fimmu.2025.1528707)

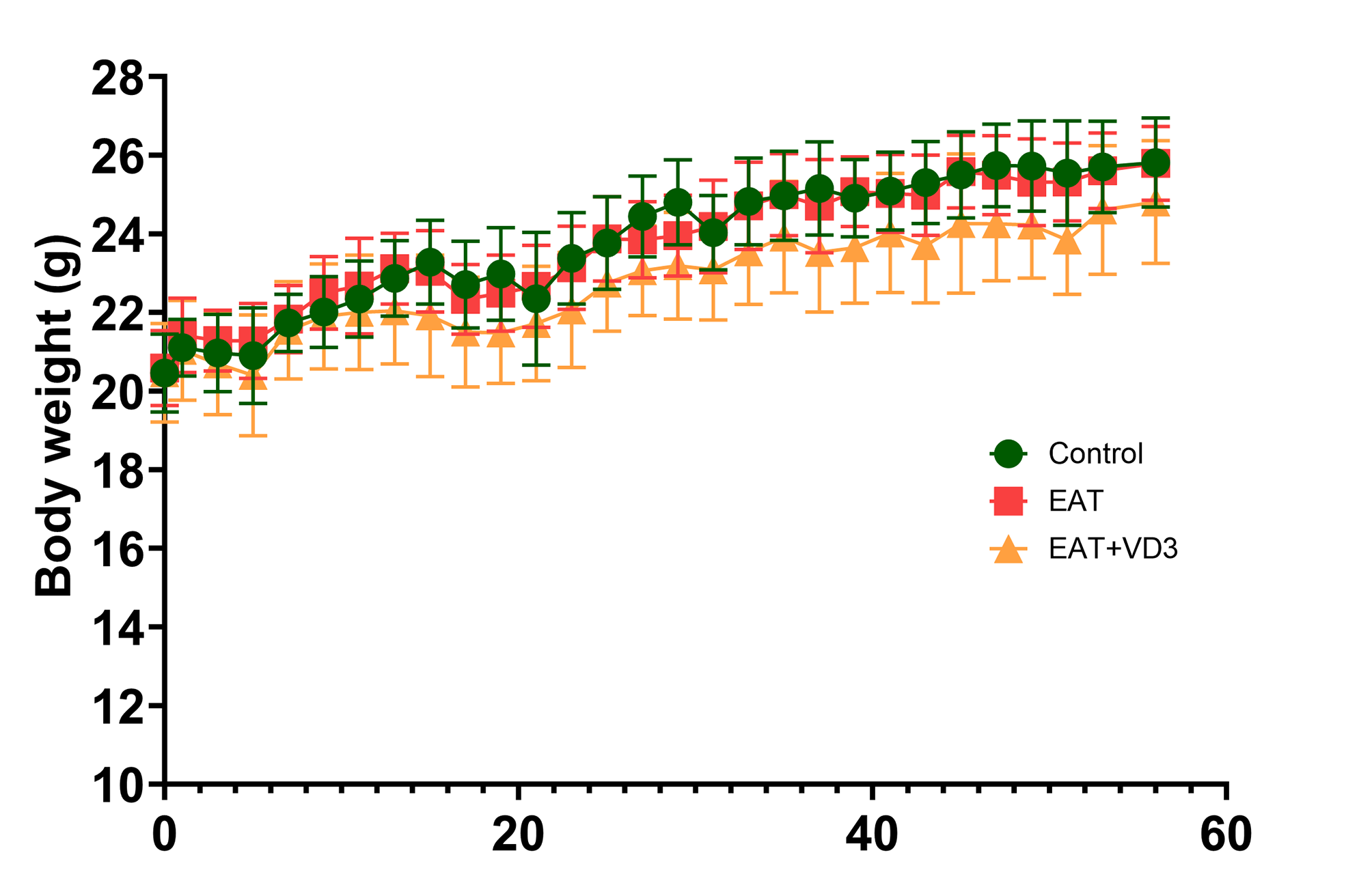

Supplement: Supplementary Figure 1 — Weight-age curves were documented for the control group, the EAT group, and the EAT+VD3 group. There was no significant difference among the three groups of experimental animals. n=16. [file Image1.tif]
